# *Urtica dioica* (Stinging Nettle): A Neglected Plant With Emerging Growth Promoter/Immunostimulant Properties for Farmed Fish

**DOI:** 10.3389/fphys.2018.00285

**Published:** 2018-03-26

**Authors:** Gionata De Vico, Vincenzo Guida, Francesca Carella

**Affiliations:** ^1^Department of Biology, University of Naples Federico II, Naples, Italy; ^2^Calatia Agricultural Society, Caserta, Italy

**Keywords:** *Urtica dioica*, growth promoter, immunostimulant, farmed fish, nettle

## Abstract

*Urtica dioica* (stinging nettle), is a perennial plant belonging to the family of *Urticaceae*, genus *Urtica*. Despite the use of nettle in folk veterinary medicine is well documented, *U. dioica* is today an underestimated and frequently neglected plant, considered by the contemporary agriculture as a weed to be eliminated. This mini review focus on very recent studies on dietary administration of *U. dioica*, both as a single herb or in combination with other herbs, to enhance growth and stimulate farmed fish immunity, thus enabling the fish to be more resistant against bacterial infections. Such an emerging feature, together with cost-effectiveness, adequate availability, and easy processing of nettle, could make this herb an excellent, inexpensive and widely used dietary supplement on intensive fish farms.

## Introduction

Immunostimulants and growth promoters consisting of herbs or their extracts, are frequently administered to farmed fishes (Harikrishnan et al., 2011; Chakraborty et al., 2013; Van Hai, 2015). In fact, when added to diet, they have proven to be effective in a dose dependent manner in stimulating their immune system and improving growth performances (Harikrishnan et al., 2011; Chakraborty et al., 2013; Van Hai, 2015; Vallejos-Vidal et al., 2016). According to Reverter et al. (2017) plants belonging to the order Lamiales (family *Lamiaceae*) are the most studied for their application in aquaculture, and had the highest number of species displaying immunostimulant activity. However, *U. dioica*, a polyvalent plant belonging to the order Rosales, that has a long history of traditional medicinal uses in many countries in the world (Ahmed and Parasuraman, 2014), seems to possess unexpected biological properties useful also for aquaculture. Such an emerging feature, together with cost-effectiveness, adequate availability, and easy processing, could make this herb an excellent, inexpensive and widely used dietary supplement on intensive farms. Unfortunately, despite its great potentials as multi-purpose crop under a low input cultivation, *U. dioica* is today an underestimated and frequently neglected plant, considered by the contemporary agriculture as a weed to be eliminated (Di Virgilio et al., 2015). Furthermore, the literature concerning its use in fishes is still fragmentary, which could limit the effort for studying the application of this plant in aquaculture. This mini review focus on very recent studies on dietary administration of *U. dioica*, both as a single herb or in combination with other herbs, to enhance growth and stimulate farmed fish immunity, thus enabling the fish to be more resistant against bacterial infections.

## Botanical pharmacology of *U. dioica*

*Urtica dioica* (stinging nettle), is a perennial plant belonging to the genus *Urtica* of the family *Urticaceae* (Ahmed and Parasuraman, 2014). The stem is erect and green, quadrangular, with lacunar collenchyma at each corner. Fibrovascular bundles could be 12–20 (Corsi and Masini, 1997). The leaves are dark-green above and paler beneath, oblong or ovate, opposite, cordate at the base, finely toothed (Testai et al., 2002). Stinging trichomes cover both stems and leaves, and contain a fluid enriched in histamine, acetylcholine, and serotonin (Tuberville et al., 1996). The small dioecious flowers are either male or female in separate inflorescences, brown to greenish in color, occurring as racemes in the axils of the upper leaves and flowering from May to September every year (Corsi and Masini, 1997; Ahmed and Parasuraman, 2014). A rhizome is present and the root is usually biarch (Corsi and Masini, 1997). Flavonoids, tannins, volatile compounds, fatty acids, polysaccharides, isolectins, sterols, terpenes, protein, vitamins, and minerals are among the main chemical constituents of *U. dioica* (Joshi et al., 2014). In this context, flavonoids and caffeic acid derivatives contribute to the anti-inflammatory, antioxidant and analgesic activities of *Urtica* leaves extracts **(**Chrubasik et al., 2007a). In particular, the anti-inflammatory effect of ethanolic extracts of *U. dioica leaves* is caused by inhibition of NF-kB pathway, which ultimately regulates inflammatory cytokine release (Chrubasik et al., 2007a). On the other hand, patient with benign prostatic hyperplasia (BPH) may benefit from nettle *root* extracts treatment thanks to at least three different mechanisms: (1) Lignans from an aqueous nettle root extract that inhibit the binding of Sex Hormone Binding Globulin (SHBG) to receptors on human prostatic membranes, which are involved in BPH pathogenesis (Chrubasik et al., 2007b; EMA, 2012); (2) chemicals found in a heptane fraction of *U. dioica* extracts inhibited aromatization of androstenedione, thus interfering with the conversion of testosterone into estrogen, a well-known mechanism in BPH pathogenesis (Chrubasik et al., 2007b; EMA, 2012); and (3) inhibition of aromatase gene expression may also be involved in the nettle root effect (Chrubasik et al., 2007b; EMA, 2012). The immunomodulating activity of *U. dioica* seems to be ascribable to its polysaccharide and lectin fractions, able to stimulate proliferation and interferon secretion of human lymphocytes (EMA, 2012). Finally, from a nutritional point of view, *U. dioica* leaves contain considerable amounts of essential amino acids, essential fatty acids, minerals, and vitamins (Rutto et al., 2013).

## Ethnoveterinary use of *U. dioica*

The use of nettle in folk veterinary medicine is well documented and it is difficult to give an exhaustive view in this short paper. Briefly, in Europe, ethno-veterinary reports documented the use of *U. dioica* at least in Italy, Switzerland, Spain, and Austria (Disler et al., 2014). In Italy and Spain *U. dioica* was administered to chickens and turkeys as growth promoter and to stimulate hens to lay (Viegi et al., 2003; Bonet and Valles, 2007; Benítez et al., 2012). In some Italian regions, pigs were fed with boiled nettle to improve their resistance to infectious diseases; furthermore, *U. dioica* tips along with *Malva sylvestris* were also given to cows as a decoction after calving (Viegi et al., 2003). In Switzerland, stinging nettle orally administered to animals (both as raw material and infusion), improves general strengthening and is used for curing genital, gastrointestinal, skin and metabolic disorders (Disler et al., 2014). *U. dioica* is also used in traditional veterinary medicine in Canada, where it is given to ruminant to improve fertility and in pregnant and lactating ones to provide trace minerals and as tonic (Lans et al., 2007). In India, animal disorders, such as haematuria, rheumatism, neck sore, infertility, bone fracture, wounds, sprains, lactation, abdominal pain, and internal injury, are also cured with nettle (Pande et al., 2007).

## *U. dioica* as growth promoter and immunostimulant in fish

Bacterial infections are considered the major cause of growth retardation and/or mortality in aquaculture for which prevention is the most important measure (Madhuri et al., 2012).

Most of the studies concerning the use of *U. dioica* as growth promoter and immunostimulant in fish, involved species of either economic interest, such as *Oncorhynchus mykiss*, or endangered (*Labeo victorianus, Huso huso*) and even ornamental fish (*Carassius auratus*).

### *Urtica dioica* as immunostimulant and growth promoter in *O. mykiss*

Major producing countries of *O. mykiss* include Chile, Italy, Norway, France, Spain, Denmark, Germany, Iran, USA, and the UK. In 2015 the global aquaculture production of *O. mykiss* reached about 762,000 tons (FAO, 2017).

According to Awad and Austin (2010) feeding Rainbow trout with a diet including 1% stinging nettle significantly reduce mortalities after challenge with *Aeromonas hydrophila*. Furthermore, in the group fed *U. dioica* they also reported an increase in values of haematocrit, hemoglobin, antiprotease, total protein, serum bactericidal activity, respiratory burst, myeloperoxidase, complement, and lysozyme activity (Awad and Austin, 2010).

More recently, Saeidi asl et al. (2017) demonstrated that *O. mykiss* juveniles receiving 3% *U. dioica* dietary supplementation improved weight gain, growth rate and feed conversion ratio. Furthermore, hematological responses including: haematocrit (Htc), hemoglobin (Hb), lymphocyte, neutrophil populations, and total red blood cells, mucus bactericidal activity, significantly increased after 8 weeks in the nettle group (Saeidi asl et al., 2017). In the same experiment the cumulative mortality of rainbow trout juveniles subjected to *Yersinia ruckeri* infection exhibited a significantly low mortality levels in *U. dioica* group compared to controls, suggesting that dietary administration of *U. dioica* enhanced growth and stimulated fish immunity (Saeidi asl et al., 2017).

Awad et al. (2012) also demonstrated that feeding rainbow trout for 2 months with a diet containing 1–2% of a mixture of herbs (*Lupinus perennis, Mangifera indica*, and *U. dioica*) resulted in weight gain, fish length and growth rate significantly higher in treated fish than in control ones. Immune parameters such as lysozyme, antiprotease, total protein, myeloperoxidase, bactericidal activity, and IgM titers were also enhanced by adding 1% nettle extract (Quercetin) to trout diet (Awad et al., 2013).

Bilen et al. (2016), fed three different groups of trout for 30 days with diets containing three different concentrations of a nettle extract. At the end of the feeding trial, rainbow trout were challenged with *A. hydrophila*, resulting in a fish weight, growth rate, and survival rate higher in treated groups than in control group (Bilen et al., 2016). Furthermore, all measured immune parameters improved after dietary administration of nettle extract, with a significantly higher level of phagocytic, lysozyme and myeloperoxidase activity compared to control (Bilen et al., 2016).

### *Urtica dioica* studies in *Labeo victorianus* and *Beluga sturgeon* (*Huso huso*), two “critically endangered species” of economic and social relevance

Currently, *L. victorianus* and *Beluga sturgeon (H. huso)* are both listed at the International Union for Conservation of Nature (IUCN) as “critically endangered species” (Maithya et al., 2005; Gesner et al., 2010; FishBase team RMCA Geelhand, 2016).

As far as *L. victorianus* is concerned, before the 1950s, it was a fished species of major commercial value in Lake Victoria (Ogutu-Ohwayo, 1990; Kembenya et al., 2017). Unfortunately, a combination of factors, including illegal fishing, overfishing, as well as competition with invasive alien species, led to a rapid collapse of the population in the late 1950s (Ogutu-Ohwayo, 1990; Kembenya et al., 2017). Currently, Kenya government supports aquaculture projects of this species in attempt to reduce the exploitation of residual natural populations and promote restocking (Kembenya et al., 2017). In this context, juveniles and adults of *L. victorianus* were challenged against *A. hydrophila* to assess the effect on growth performance and immune parameters of 5% *U. dioica* dietary supplementation of for 16 weeks (Ngugi et al., 2015). In particular, serum immunoglobulins, lysozyme activity and respiratory burst significantly increased, with fish survival reaching up to 95% after the bacterial challenge, a percentage significantly higher compared to the control group (Ngugi et al., 2015). Furthermore, adding nettle to diet, increased growth performance, hematological (red blood cell count [RBC], white blood cell counts [WBC], haematocrit [Hct], mean cell hemoglobin concentration [Hb]), biochemical (total serum protein and albumin), and immunological parameters in experimental fishes (Ngugi et al., 2015).

Historical data reported that the *Beluga sturgeon* (*H. huso*) used to live in the Pô river (Italy), in the Adriatic Sea and in the Danube river (Billard and Lecointre, 2001). In the last 40 years, stocks of sturgeons are dramatically decreasing due to overfishing and environmental degradation (Billard and Lecointre, 2001). Because of this decline, sturgeon aquaculture have been developed which hopefully may contribute to reduce fishing pressure and increase wild stocks (Bronzi et al., 2011). However, development of sturgeon farming was also accompanied by disease outbreaks, including those unknown prior to cultivation (Bauer et al., 2002). For that reason, lately, increased attention has been directed toward the use of immunostimulant in *Beluga sturgeon* aquaculture, in order to improve fish health (Jalali et al., 2009).

In this context, Binaii et al. (2014) found that feeding beluga juveniles for 8 weeks with increasing amount of nettle in the diet (0–12%) resulted in a significant increase in neutrophils and Hb levels compared to the controls. Furthermore, the increase of the above parameters was dose-dependent, with 12% nettle group showing the highest Hb, RBC, WBC, Hct, respiratory burst activity, total immunoglobulin, and total protein values (Binaii et al., 2014). Other studies of Nobahar et al. (2015) the effect of 1% *U. dioica* diet supplementation on juveniles of *Beluga sturgeon* with mean weight of 30 ± 0.5 g reared for resettlement. After 60 days treatment, Condition Factor, Hb concentration, Hct value, Neutrophils and Lymphocytes were significantly higher in fish fed nettle than in control group.

### Ornamental fish

Production of aquarium fish is becoming an important business activity in aquaculture because of an incessant increase in demand for ornamental fishes by hobbyists from all over the world (Yanar et al., 2008). Unfortunately, as with other types of breeding, one of the major limiting factors is infectious and parasitic diseases (Kumar et al., 2013). In this regard, Bilen et al. (2014) demonstrated that supplementing diet with 0.5 g/kg of a methanolic extract of *U. dioica* for 30 days is sufficient to increase non-specific immune response of gold fish (*C. auratus*) with an increased superoxide anion production, lysozyme, myeloperoxidase, and phagocytic activity.

## Conclusion

Among the food production sectors, aquaculture is the fastest growing in the world, and is expected to be the biggest source of food by the year 2030 (Brugère and Ridler, 2004; Carella and Sirri, 2017). Such an increase in fish farming, results in a parallel increase in outbreaks of infectious and metabolic diseases, which act as major limiting factors for aquaculture development (Wunderlich et al., 2017). In order to control mortality and avoid huge economic losses, fish farmers frequently adopt inappropriate practices, which disregard care for the environmental, animal and human health. Among them, the indiscriminate use of pesticides, disinfectants and antibiotics is of major concern (Valladão et al., 2015). For the above reasons, attention to a much more “natural” approach in aquaculture development is increasing worldwide (Xie et al., 2013). According to organic standards, health of the cultivated organisms shall be mainly guaranteed through preventive measures (i.e., optimizing management, feed, and diet). However, studies suggested that the use of disinfectants and antibiotics are not always effective in prevention or control diseases in aquaculture (Kumar et al., 2013). For that reason, to improve fish immunity and increase resistance to infectious diseases, immunostimulant are today routinely administered in fish farming (Kumar et al., 2013; Nobahar et al., 2015). However, the high cost of such additives and their impact on ecosystem health, risks becoming deterrent to their use, and the search for cheaper and more natural compounds is an urgent necessity for modern aquaculture. In this context, large numbers of plants have been proven to be rich sources of cheaper immune-enhancing and growth promoter substances, with a wide therapeutic and preventive spectrum of activity, potentially useful in solving the multiple health problems that characterize aquaculture (Karatas et al., 2003; Kumar et al., 2013). According to our knowledge, the effects of dietary nettle as growth promoter and immunostimulant in fish were examined in this brief survey. The review of the literature, thought limited, provides evidence for the effective immunostimulant use of nettle in aquaculture, and open new perspective for the use of *U. dioica* as cost-effective adjuvant therapy added to fish food to prevent diseases and increase growth. However, the biological properties reviewed in this paper, perhaps represent only the tip of the iceberg of possible uses of nettle in aquaculture: some recent studies show, for example, the great efficacy of nettle extracts against the main pathogenic bacteria for fish in aquaculture (Dar et al., 2012). Thus, it is hopeful that further studies involving determination of optimal doses, the active principle of the plant extract and feeding protocols for food additives could led to widespread application of *U. dioica* in current aquaculture.

## Author contributions

All authors listed have made a substantial, direct and intellectual contribution to the work, and approved it for publication.

### Conflict of interest statement

The authors declare that the research was conducted in the absence of any commercial or financial relationships that could be construed as a potential conflict of interest.

## References

[B1] Ahmed M. K. K. Parasuraman S. (2014). *Urtica dioica* L., (Urticaceae): a stinging nettle. Syst. Rev. Pharm. 5, 6–8. 10.5530/srp.2014.1.3

[B2] Awad A. Austin B. (2010). Use of lupin *(Lupinus perennis)*, mango *(Mangifera indica)* and stinging nettle *(Urtica dioica)* as feed additives to prevent *Aeromonas hydrophila* infection in rainbow trout *(Oncorhynchus mykiss*, Walbaum). J. Fish Dis. 33, 413–420. 10.1111/j.1365-2761.2009.01133.x 20102439

[B3] Awad E. Austin B. Lyndon A. (2012). Effect of dietary supplements on digestive enzymes and growth performance of rainbow trout (*Oncorhynchus mykiss*, Walbaum). J. Am. Sci. 8, 858–864. 10.7537/marsjas081212.119

[B4] Awad E. Austin D. Lyndon A. R. (2013). Effect of black cumin seed oil (*Nigella sativa*) and nettle extract (Quercetin) on enhancement of immunity in rainbow trout, *Oncorhynchus mykiss* (Walbaum). Aquaculture 388–391, 193–197. 10.1016/j.aquaculture.2013.01.008

[B5] Bauer O. N. Pugachev O. N. Voronin V. N. (2002). Study of parasites and diseases of sturgeons in Russia: a review. J. Appl. Ichthyol. 18, 420–429. 10.1046/j.1439-0426.2002.00422.x

[B6] Benítez G. González-Tejero M. R. Molero-Mesa J. (2012). Knowledge of ethnoveterinary medicine in the Province of Granada, Andalusia, Spain. J. Ethnopharmacol. 139, 429–439. 10.1016/j.jep.2011.11.029 22155471

[B7] Bilen S. Ünal S. Güvensoy H. (2016). Effects of oyster mushroom (*Pleurotus ostreatus*) and nettle (*Urtica dioica*) methanolic extracts on immune responses and resistance to *Aeromonas hydrophila* in rainbow trout (*Oncorhynchus mykiss*). Aquaculture 454, 90–94. 10.1016/j.aquaculture.2015.12.010

[B8] Bilen S. Soydas E. Bilen A. M. (2014). Effects of methanolic extracts of nettle (*Urtica dioica*) on non-specific immune response of gold fish (*Carassius auratus*). Alinteri J. Agric. Sci. 27, 24–28.

[B9] Billard R. Lecointre G. (2001). Biology and conservation of sturgeon and paddlefish. Rev. Fish Biol. Fish. 10, 355–392. 10.1023/A:1012231526151

[B10] Binaii M. Ghiasi M. Farabi S. M. V. Pourgholam R. Fazli H. Safari R.. (2014). Biochemical and hemato-immunological parameters in juvenile beluga (*Huso huso*) following the diet supplemented with nettle (*Urtica dioica*). Fish Shell. Immun. 36, 46–51. 10.1016/j.fsi.2013.10.001 24516872

[B11] Bonet M. A. Vallès J. (2007). Ethnobotany of Montseny biosphere reserve (Catalonia, Iberian Peninsula): plants used in veterinary medicine. J. Ethnopharmacol. 110, 130–147. 10.1016/j.jep.2006.09.016 17059874

[B12] Bronzi P. Rosenthal H. Gessner J. (2011). Global sturgeon aquaculture production. J. Appl. Ichthyol. 27, 169–175. 10.1111/j.1439-0426.2011.01757.x

[B13] Brugère C. Ridler N. (2004). Global Aquaculture Outlook in the Next Decades: An Analysis of National Aquaculture Production Forecasts to 2030. FAO Fisheries Circular No. 1001. Rome: FAO.

[B14] Carella F. Sirri R. (2017). Editorial: fish and shellfish pathology. Front. Mar. Sci. 4:375 10.3389/fmars.2017.00375

[B15] Chakraborty S. B. Horn P. Hancz C. (2013). Application of phytochemicals as growth-promoters and endocrine modulators in fish culture. Rev. Aquacult. 5, 1–19. 10.1111/raq.12021

[B16] Chrubasik J. E. Roufogalis B. D. Wagner H. Sigrun A. Chrubasik S. A. (2007a). A Comprehensive review on nettle effect and efficacy profiles, Part I: herba urticae. Phytomedicine. 14, 423–435. 10.1016/j.phymed.2007.03.004 17493795

[B17] Chrubasik J. E. Roufogalis B. D. Wagner H. Chrubasik A. (2007b). Comprehensive review on the stinging nettle effect and efficacy profiles. Part II: Urticae radix. Phytomedicine 14, 568–579. 10.1016/j.phymed.2007.03.014 17509841

[B18] Corsi G. Masini A. (1997). Anatomical and ecological aspect in Italian taxa of the genus ortica. Atti Soc. Tosc. Sci. Nat. Mem. Ser. B 104, 1–8.

[B19] Dar S. A. Ganai F. A. Yousuf A. R. Balkhi M. H. Bhat T. M. Bhat F. A. (2012). Bioactive potential of leaf extracts from *Urtica dioica* L. against fish and human pathogenic bacteria. Afr. J. Microbiol. Res. 6, 6893–6899. 10.5897/AJMR12.336

[B20] Disler M. Ivemeyer S. Hamburger M. Vogl C. R. Tesic A. Klarer F.. (2014). Ethnoveterinary herbal remedies used by farmers in four north-eastern Swiss cantons (St. Gallen, Thurgau, Appenzell Innerrhoden and Appenzell Ausserrhoden). J. Ethnobiol. Ethnomed. 10:32. 10.1186/1746-4269-10-32 24685062 PMC4022237

[B21] Di Virgilio N. Papazoglou E. G. Jankauskiene Z. Di Lonardo S. Praczyk M. Wielgusz K. (2015). The potential of stinging nettle (*Urtica dioica* L.) as a crop with multiple uses. Indust. Crops Prod. 68, 42–49. 10.1016/j.indcrop.2014.08.012

[B22] European Medical Agency (EMA) (2012). Assessment Report on Urtica dioica L., Urtica urens L., Their Hybrids or Their Mixtures, Radix. Available on line at: http://www.ema.europa.eu/docs/en_GB/document_library/Herbal_-_HMPC_assessment_report/2012/11/WC500134484.pdf

[B23] FAO (2017). Fishery and Aquaculture Statistics. FAO Yearbook of Fisheries Statistics 2015. Food and Agriculture Organization of the United Nations Available online at: http://www.fao.org/3/a-i7989t.pdf

[B24] FishBase team RMCA and Geelhand D. (2016). Labeo victorianus. The IUCN Red List of Threatened Species. e.T60318A47182908. 10.2305/IUCN.UK.2016-3.RLTS.T60318A47182908.en

[B25] Gesner J. Chebanov M. Freyhof J. (2010). Huso huso. The IUCN Red List of ThreatenedSpecies. e.T10269A3187455. 10.2305/IUCN.UK.2010-1.RLTS.T10269A3187455.en

[B26] Harikrishnan R. Balasundaram C. Heo M.-S. (2011). Impact of plant products on innate and adaptive immune system of cultured finfish and shellfish. Aquaculture 317, 1–15. 10.1016/j.aquaculture.2011.03.039

[B27] Jalali M. A. Ahmadifar E. Sudagar M. Takami G. A. (2009). Growth efficiency, body composition, survival and haematological changes in great sturgeon (*Huso huso* Linnaeus, 1758) juveniles fed diets supplemented with different levels of Ergosan. Aquacult. Res. 40, 804–809. 10.1111/j.1365-2109.2009.02166.x

[B28] Joshi B. C. Mukhija M. Kalia A. N. (2014). Pharmacognostical review of *Urtica dioica* L. Int. J. Green Pharm. 8, 201–209. 10.4103/0973-8258.142669

[B29] Karatas S. Dügenci N. Akin A. (2003). Some medicinal plants as immunostimulant for fish. Can. J. Ethnopharmacol. 88, 99–106. 10.1016/S0378-8741(03)00182-X 12902058

[B30] Kembenya E. M. Marcial H. S. Nicholas O. Outa N. O. Sakakura Y. Hagiwara A. (2017). Captive breeding of threatened African Carp, *Labeo victorianus*, of Lake Victoria. J. World Aquacult. Soc. 48, 955–962. 10.1111/jwas.12328

[B31] Kumar S. Raman R. P. Pandey P. K. Mohanty S. Kumar A. Kumar K. (2013). Effect of orally administered azadirachtin on non-specific immune parameters of goldfish *Carassius auratus* (Linn. 1758) and resistance against *Aeromonas hydrophila*. Fish Shell. Immunol. 34, 564–573. 10.1016/j.fsi.2012.11.038 23261511

[B32] Lans C. Turner N. Khan T. Brauer G. Boepple W. (2007). Ethnoveterinary medicines used for ruminants in British Columbia. Can. J. Ethnobiol. Ethnomed. 3:11. 10.1186/1746-4269-3-11 17324258 PMC1831764

[B33] Madhuri S. Mandloi A. K. Govind P. Sahni Y. P. (2012). Antimicrobial activity of some medicinal plants against fish patholgens. Int. Res. J. Pharm. 3, 28–30.

[B34] Maithya J. Charo H. Okeyo-Owuor J. B. Wangila B. C. C. Ouma H. Orinda C. (2005). Aquaculture strategy for restoration of threatened Lake Victoria fishes: the case for *Oreochromis variabilis* (Boulenger, 1906) and *Labeo victorianus* (Boulenger, 1901), in Knowledge and Experiences Gained From Managing the Lake Victoria Ecosystem, eds Mallya G. A. Katagira F. F. Kanggoha G. Mbwana S. B. Katunzi E. F. Wambende J. T. Azza N. Wakwabi E. Njoka S. W. Kusewa M. Busulwa H. (Dar es Salaam: Lake Victoria Environmental Management Project), 445–466.

[B35] Ngugi C. C. Oyoo-Okoth E. Mugo-Bundi J. Orina P. S. Chemoiwa E. J. Aloo P. A. (2015). Effects of dietary administration of stinging nettle (*Urtica dioica*) on the growth performance, biochemical, hematological and immunological parameters in juvenile and adult Victoria Labeo (*Labeo victorianus*) challenged with *Aeromonas hydrophila*. Fish. Shellfish Immunol. 44, 533–541. 10.1016/j.fsi.2015.03.025 25827627

[B36] Nobahar Z. H. Gholipour-Kanani H. Kakoolaki S. H. Jafaryan H. (2015). Effect of garlic (*Allium sativum*) and nettle (*Urtica dioica*) on growth performance and hematological parameters of beluga (*Huso huso*). Iran. J. Aquat. Anim. Health 1, 63–69. 10.18869/acadpub.ijaah.1.1.63

[B37] Ogutu-Ohwayo R. (1990). The decline of the native fishes of Lakes Victoria and Kyoga (East Africa) and the impact of introduced species, especially the Nile perch, *Lates niloticus* and the Nile tilapia, *Oreochromis niloticus*. Environ. Biol. Fish. 27, 81–90. 10.1007/BF00001938

[B38] Pande P. C. Lalit Tiwari L. Pande H. C. (2007). Ethnoveterinary plants of Uttaranchal—A review. Ind. J. Trad. Know. 6, 444–458.

[B39] Reverter M. Tapissier-Bontemps N. Sasal P. Saulnier D. (2017). Use of medicinal plants in aquaculture, in Diagnosis and Control of Diseases of Fish and Shellfish, 1st Edn, eds Austin B. Newaj-Fyzul A. (Chichester, UK: John Wiley & Sons Ltd), 223–261. 10.1002/9781119152125.ch9

[B40] Rutto L. K. Xu Y. Ramirez E. Brandt M. (2013). Mineral properties and dietary value of raw and processed stinging nettl*e (Urtica dioica L*.). Int. J. Food Sci. 2013:857120. 10.1155/2013/857120 26904610 PMC4745470

[B41] Saeidi Asl M. R. Adel M. Caipang C. M. A. Dawood M. A. O. (2017). Immunological responses and disease resistance of rainbow trout (*Oncorhynchus mykiss*) juveniles following dietary administration of stinging nettle (*Urtica dioica*). Fish Shell. Immunol. 71, 230–238. 10.1016/j.fsi.2017.10.016 29017944

[B42] Testai L. Chericoni S. Calderone V. Nencioni G. Nieri P. Morell I.. (2002). Cardiovascular effects of *Urtica dioica* L. (Urticaceae) roots extracts*: in vitro* and *in vivo* pharmacological studies. J. Ethnopharmacol. 81, 105–109. 10.1016/S0378-8741(02)00055-7 12020933

[B43] Tuberville T. D. Dudley P. G. Pollard A. J. (1996). Responses of invertebrate herbivores to stinging trichomes of *Urtica dioica* and *Laportea canadensis*. Oikos 75, 83–88. 10.2307/3546324

[B44] Valladão G. M. Gallani S. U. Pilarski F. (2015). Phytotherapy as an alternative for treating fish disease. J. Vet. Pharmacol. Ther. 38, 417–428. 10.1111/jvp.12202 25620601

[B45] Vallejos-Vidal E. Reyes-López F. Teles M. MacKenzie S. (2016). The response of fish to immunostimulant diets. Fish Shell. Immunol. 56, 34–69. 10.1016/j.fsi.2016.06.028 27389620

[B46] Van Hai N. (2015). The use of medicinal plants as immunostimulants in aquaculture: a review. Aquaculture 446, 88–96. 10.1016/j.aquaculture.2015.03.014

[B47] Viegi L. Pieroni A. Guarrera P. M. Vangelisti R. (2003). A review of plants used in folk veterinary medicine in Italy as basis for a databank. J. Ethnopharmacol. 89, 221–224. 10.1016/j.jep.2003.08.003 14611886

[B48] Wunderlich A. C. de Oliveira Penha Zica E. dos Santos Ayres V. F. Guimarães A. C. Takeara R. (2017). Plant-derived compounds as an alternative treatment against parasites. in fish farming: a review, in Natural Remedies in the Fight Against Parasites, eds Khater H. Govindarajan M. Benelli G. (InTech), 246 10.5772/67668 Available online at: https://www.intechopen.com/books/natural-remedies-in-the-fight-against-parasites/plant-derived-compounds-as-an-alternative-treatment-against-parasites-in-fish-farming-a-review

[B49] Xie B. Qin J. Yang H. Wang X. Wang Y.-H. Li T. (2013). Organic aquaculture in China: a review from a global perspective. Aquaculture 414–415, 243–253. 10.1016/j.aquaculture.2013.08.019

[B50] Yanar M. Erçen Z. Hunt A. Ö. Büyükçapar H. M. (2008). The use of alfalfa, *Medicago sativa* as a natural carotenoid source in diets of goldfish, *Carassius auratus*. Aquaculture 284, 196–200. 10.1016/j.aquaculture.2008.07.050

